# Supplementary material for: One-Pot Environmentally Friendly Synthesis of Nanomaterials Based on Phytate-Coated Fe3O4 Nanoparticles for Efficient Removal of the Radioactive Metal Ions 90Sr, 90Y and (UO2)2+ from Water
Source: Nanomaterials (Basel). 2022 Dec 9;12(24):4383. doi: 10.3390/nano12244383 (PMC9781934; doi:10.3390/nano12244383)
Supplement: Supplementary file 1 [file nanomaterials-12-04383-s001.zip › nanomaterials-2072514-supplementary.pdf]

Supplementary Materials

One-Pot Environmentally Friendly Synthesis of Nanomaterials Based on Phytate-Coated Fe_3O_4 Nanoparticles for Efficient Removal of the Radioactive Metal Ions ^{90}Sr, ^{90}Y and $(UO_2)^{2+}$ from Water

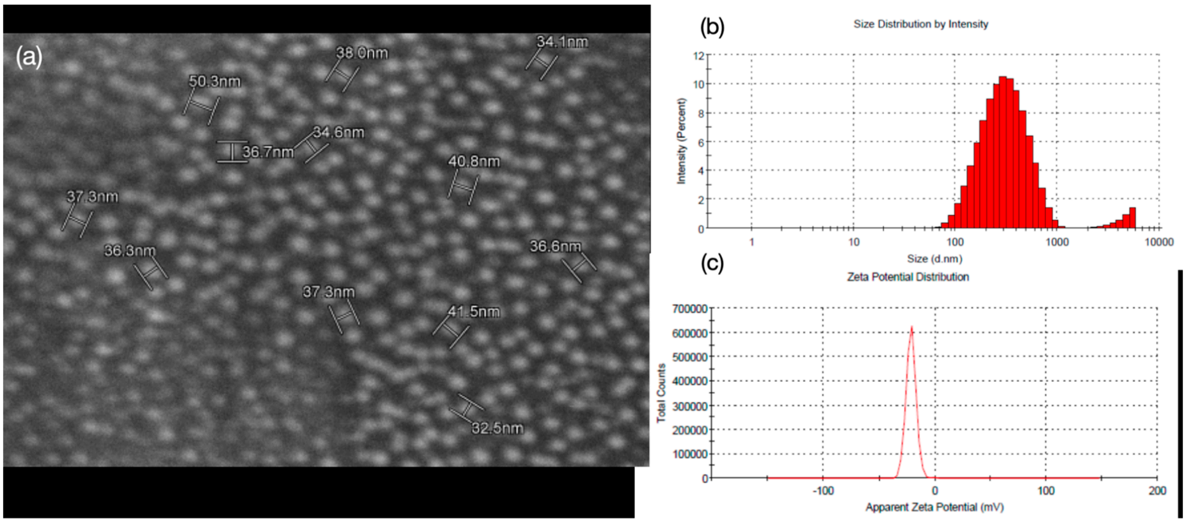

Figure S1. a. TEM micrograph, **b**. Dynamic light Scattering (DLS) analysis and **c**. Zeta Potential of Fe_3O_4 NPs**.**

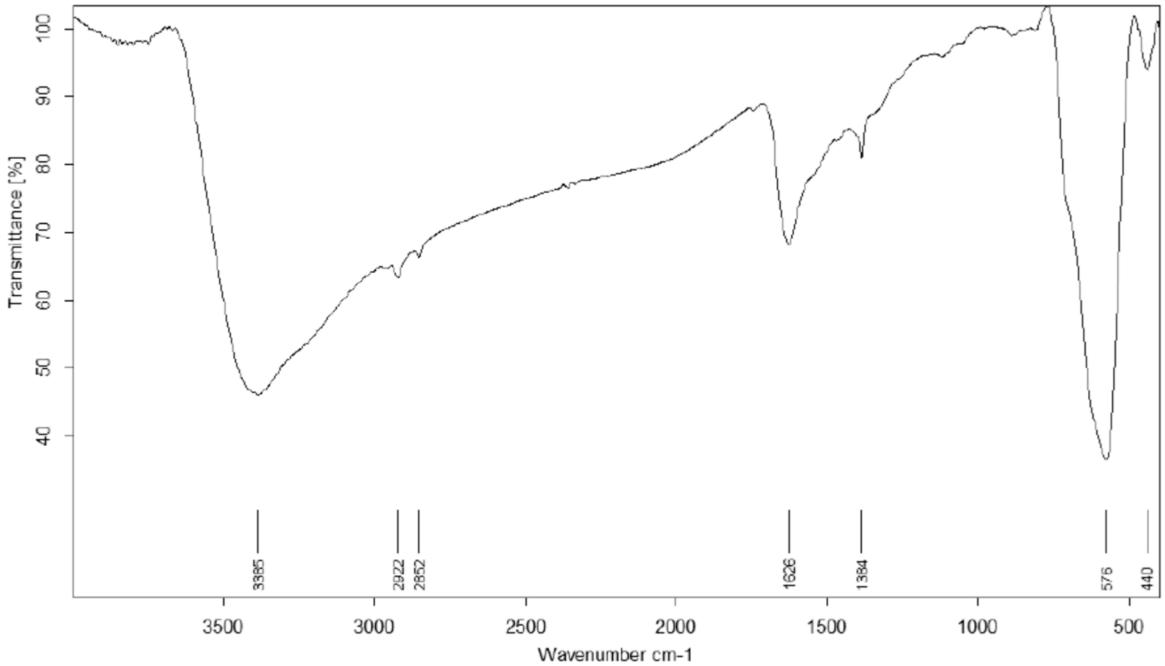

Figure S2. FT-IR spectra of pristine magnetite nanoparticles.

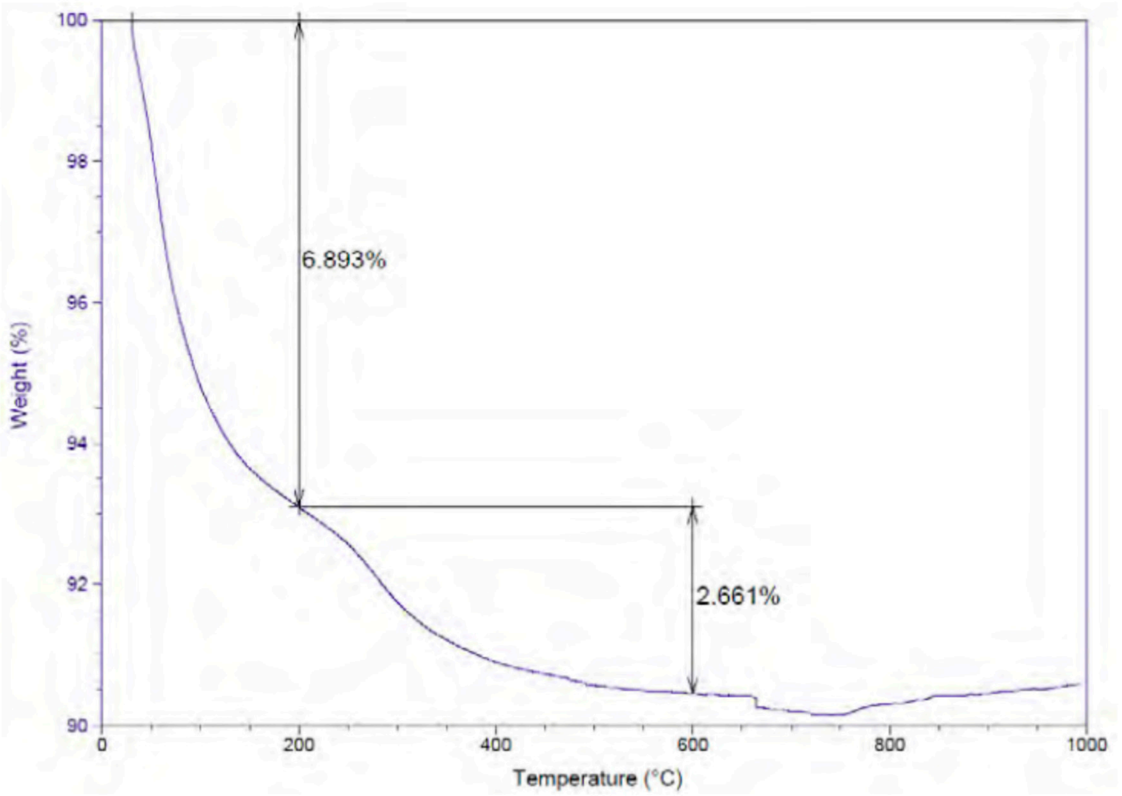

Figure S3. Thermogravimetric analysis (TGA) of pristine magnetite nanoparticles.

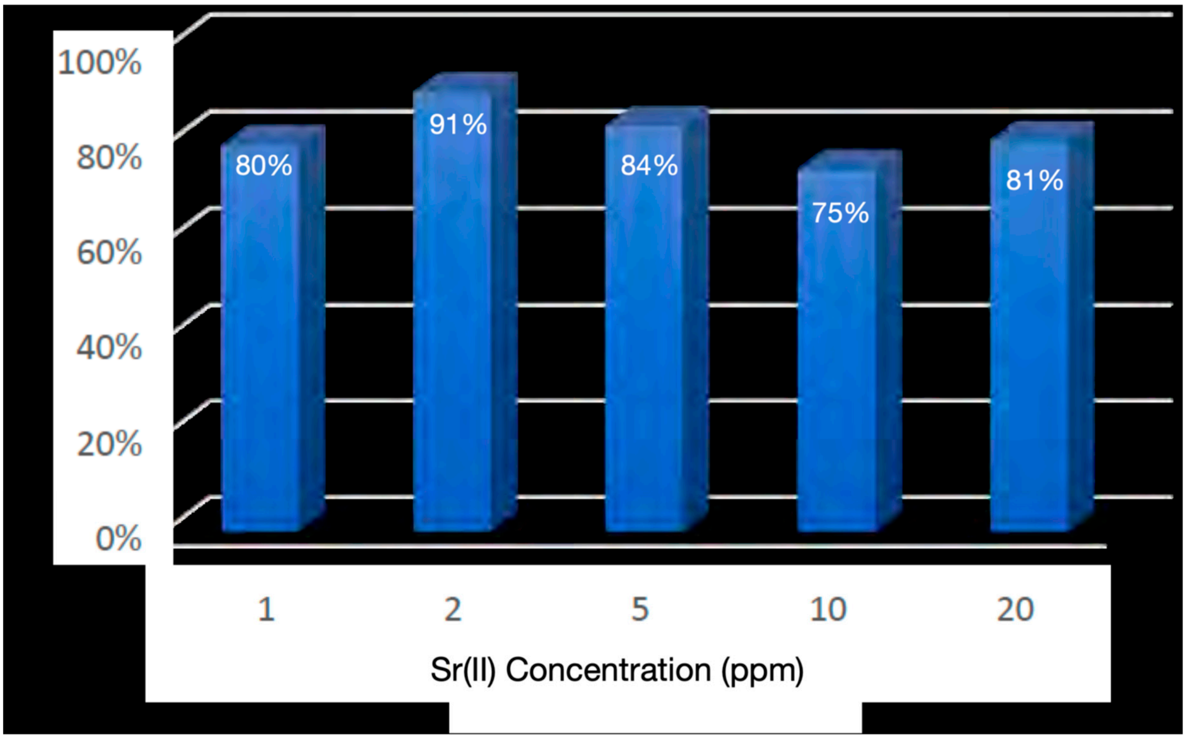

Figure S4. Retention percentage of Sr(II) by Phy@Fe_3O_4 NPs versus the initial concentration of Sr(II).

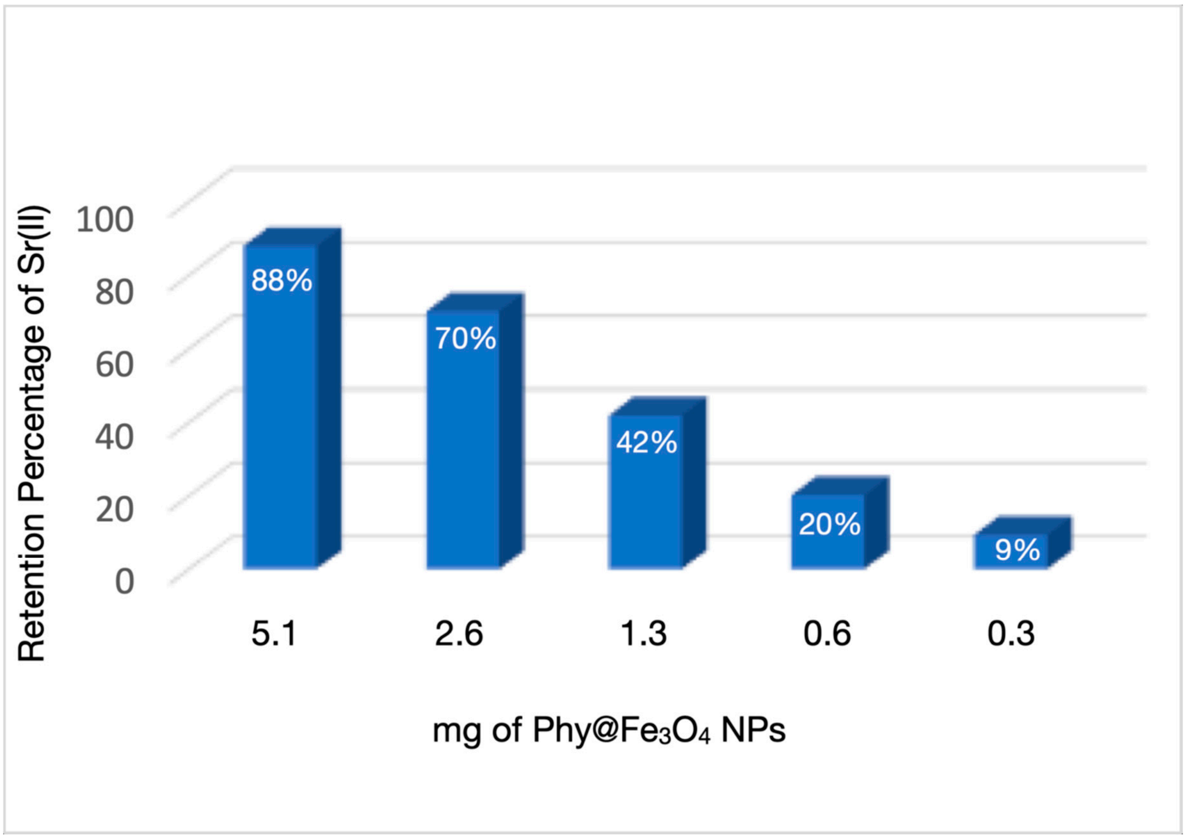

Figure S5. Sr(II) retention percentage with different amounts of Phy@Fe_3O_4 NPs.

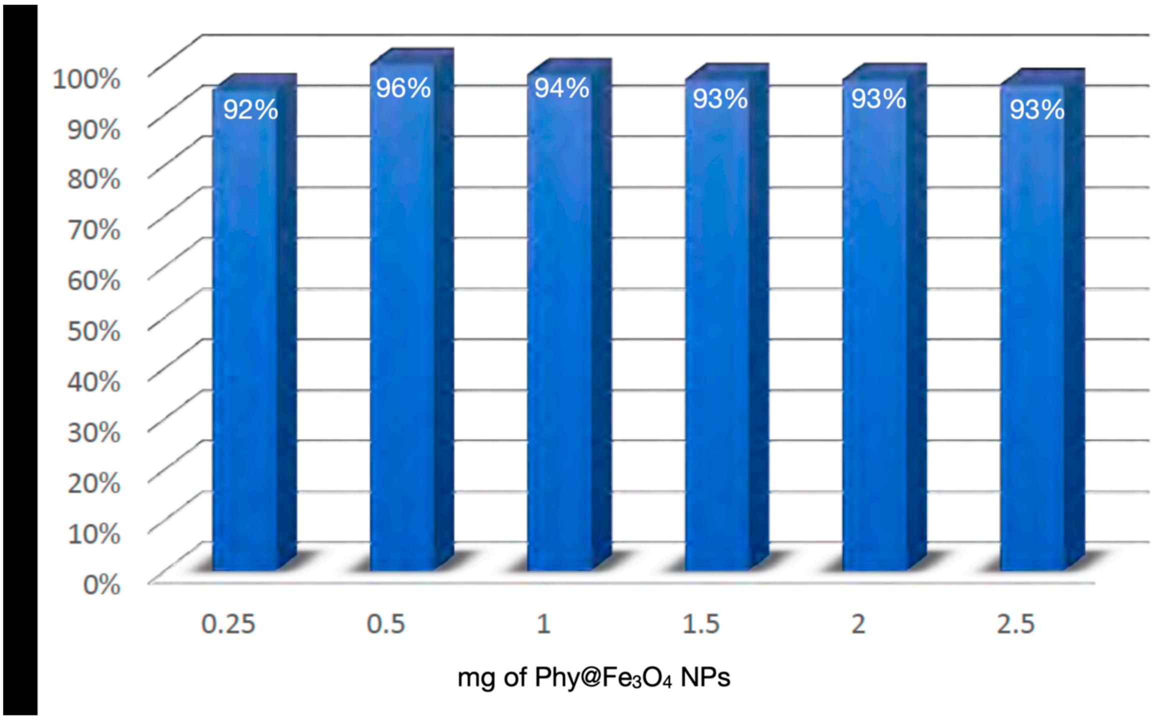

Figure S6. UO_2^{2+} retention percentage with different amounts of Phy@Fe_3O_4 NPs.
